# Concordance analysis for QTL detection in dairy cattle: a case study of leg morphology

**DOI:** 10.1186/1297-9686-46-31

**Published:** 2014-05-19

**Authors:** Irene van den Berg, Sébastien Fritz, Sabrina Rodriguez, Dominique Rocha, Mekki Boussaha, Mogens S Lund, Didier Boichard

**Affiliations:** 1 INRA, UMR1313 Génétique Animale et Biologie Intégrative, 78350 Jouy-en-Josas, France; 2 AgroParisTech, UMR1313 Génétique Animale et Biologie Intégrative, 16 rue Claude Bernard, 75231 Paris 05, France; 3 Center for Quantitative Genetics and Genomics, Department of Molecular Biology and Genetics, Faculty of Science and Technology, Aarhus University, DK-8830 Tjele, Denmark; 4 UNCEIA, 149 rue de Bercy, 75012 Paris, France; 5 INRA SIGENAE Bioinformatics Team, 31326 Castanet, France

## Abstract

**Background:**

The present availability of sequence data gives new opportunities to narrow down from QTL (quantitative trait locus) regions to causative mutations. Our objective was to decrease the number of candidate causative mutations in a QTL region. For this, a concordance analysis was applied for a leg conformation trait in dairy cattle. Several QTL were detected for which the QTL status (homozygous or heterozygous for the QTL) was inferred for each individual. Subsequently, the inferred QTL status was used in a concordance analysis to reduce the number of candidate mutations.

**Methods:**

Twenty QTL for rear leg set side view were mapped using Bayes C. Marker effects estimated during QTL mapping were used to infer the QTL status for each individual. Subsequently, polymorphisms present in the QTL regions were extracted from the whole-genome sequences of 71 Holstein bulls. Only polymorphisms for which the status was concordant with the QTL status were kept as candidate causative mutations.

**Results:**

QTL status could be inferred for 15 of the 20 QTL. The number of concordant polymorphisms differed between QTL and depended on the number of QTL statuses that could be inferred and the linkage disequilibrium in the QTL region. For some QTL, the concordance analysis was efficient and narrowed down to a limited number of candidate mutations located in one or two genes, while for other QTL a large number of genes contained concordant polymorphisms.

**Conclusions:**

For regions for which the concordance analysis could be performed, we were able to reduce the number of candidate mutations. For part of the QTL, the concordant analyses narrowed QTL regions down to a limited number of genes, of which some are known for their role in limb or skeletal development in humans and mice. Mutations in these genes are good candidates for QTN (quantitative trait nucleotides) influencing rear leg set side view.

## Background

A large number of quantitative trait loci (QTL) have been detected since the availability of genetic markers. However, the mutations that underlie such QTL have been identified only in a few cases [1]. Even reasonably fine-mapped QTL regions of around 2 Mb can still contain multiple genes with a large number of potential causative mutations. Thus, the step from QTL to causative mutations remains difficult.

The present availability of whole-genome sequence data provides new opportunities to narrow down QTL regions to causative mutations [2]. One approach to do this is to eliminate a large number of potential candidate mutations by concordance analysis, which compares the QTL status (homozygous or heterozygous) with status of polymorphisms in the QTL region across genotyped individuals. Assuming a single mutation is responsible for a QTL, an animal will be homozygous for this mutation when it is homozygous for the QTL and heterozygous when it is heterozygous for the QTL [3]. Using this principle, Karlsson et al. [4] were able to reduce the number of candidate causative mutations by 37% for a locus that affects coat colour in dogs. Although quantitative traits are influenced by several mutations rather than a single mutation, concordance between a candidate mutation and the QTL genotype can provide evidence when searching for causative mutations. For example, in a study that focused on a QTL for milk yield and composition on chromosome 6, concordant polymorphisms were found only in the *ABCG2* gene [5].

With the increasing availability of sequence data, such a concordance analysis can be done on a larger scale and could be helpful to reduce the often very large number of candidate mutations in a QTL interval. When a concordance analysis is used for all polymorphisms in a QTL region, it is necessary to set a very low probability of concordance by chance to avoid type 1 errors. The probability of concordance by chance decreases with the number of individuals with predicted statuses [3]. QTL statuses can be derived using a granddaughter design [6] but not all sequenced animals will have a sufficient number of progeny to infer QTL status accurately. A method that provides QTL status for all sequenced individuals is therefore desirable.

Rear leg side view (RLSV) is a quantitative trait recorded in dairy cattle that measures the angle of the hock. Large deviations from the average score are associated with a higher culling rate [7]. Although several QTL for RLSV have been detected [8,9], the causative mutations that underlie these QTL are unknown.

In this study, we used RLSV as an example trait to assess the effectiveness of concordance analysis to narrow down from a QTL region to candidate mutations. First, QTL regions were defined, then the QTL status was derived for a large number of individuals and a concordance analysis was performed.

## Methods

### QTL mapping

Genotypes of 3154 Holstein bulls were used for QTL mapping. These bulls were nearly all Holstein artificial insemination bulls born between 1999 and 2004, owned and progeny-tested by the five major French breeding companies. The genotypes were obtained with the Illumina Bovine SNP50 BeadChip® [10] by Labogena. Quality control included: test of cluster quality, which was performed at the genotyping laboratory level; minimum SNP call rate of 99%; Hardy Weinberg equilibrium (p < 10^-4^); minimum call rate of 98%; parentage checking. These tests, as well as imputation and phasing, were performed upstream of this study, in the routine pipeline of genomic selection. After removal of markers with a minor allele frequency below 0.05, 39 683 autosomal markers were retained for analysis. For all bulls, deregressed estimated breeding values (EBV) of RLSV were used for QTL mapping. Deregressed EBV were obtained using a procedure similar to [11], except that when computing the weight *w*_*i*_, we assumed that 100% of the genetic variance was explained by the SNPs. This leads to $w_{i}=\frac{\left(1-h^{2}\right)r_{i}^{2}}{h^{2}\left(1-r_{i}^{2}\right)}$, with $r_{i}^{2}$ being the reliability of the EBV of bull *i* from progeny information only. The expectation of the bull EBV without progeny information is the pedigree index (PI), leading to the following deregressed EBV:

$$y=\mathrm{PI}+\frac{\mathrm{EBV}-\mathrm{PI}}{r^{2}}.$$

QTL mapping was done using Bayes C [12], as implemented in the GS3 software [13] according to the following statistical model:

$$y_{i}=\mu +u_{i}+\sum_{k=1}^{K}z_{\mathrm{ik}}a_{k}+e_{i},$$

where *y*_*i*_ is the deregressed EBV for individual *i*, *μ* the overall mean, *u*_*i*_ the polygenic breeding value of individual *i*, *K* the number of markers, *z*_*ik*_ the genotype of individual *i* for marker *k*, coded 0, 1 or 2 depending on the number of copies of the second marker allele, *a*_*k*_ the additive effect of marker *k*, and *e*_*i*_ the random residual for individual *i*.

All unknown parameters were assigned prior distributions and sampled with a Monte Carlo Markov chain (MCMC) using Gibbs sampling. The MCMC was run for 180 000 iterations, with a burn-in of 20 000 iterations and a thin interval of 50. The prior used for *a*_*k*_ was a mixture distribution that equals:

$$a_{k}\left|\pi ,\sigma_{a}^{2}\right)\sim \left\{\begin{array}{c} 0\;\mathrm{with}\;\mathrm{probability}\;\pi ,\\ N\left(0,\sigma_{a}^{2}\right)\mathrm{with}\;\mathrm{probability}\left(1-\pi \right), \end{array}\right)$$

where $\sigma_{a}^{2}$ is the common marker variance and the hyper parameter π is the prior probability that the effect of marker *k* is equal to 0. Variances $\sigma_{u}^{2}$, $\sigma_{a}^{2}$ and $\sigma_{e}^{2}$ were assigned inverted chi-square distributions with *v* = 4.2 degrees of freedom and scale parameter $S^{2}=\frac{{\hat{\sigma }}^{2}\left(\nu -2\right)}{\nu }$ where ${\hat{\sigma }}^{2}$ is the prior value for $\sigma_{u}^{2}$, $\sigma_{a}^{2}$ or $\sigma_{e}^{2}$. Parameter π was fixed at 0.99, following [14].

To select QTL regions for further analyses, intervals of 40 adjacent markers (corresponding on average to 2.5 Mb) were ranked based on the sum of their posterior inclusion probabilities (∑p). The posterior inclusion probability of a marker is the proportion of iterations that included the marker in the model. Since our aim was to select the largest QTL rather than all QTL, the 20 intervals with the highest ∑p were selected and denoted as QTL. If intervals overlapped, only the interval with the highest ∑p was selected. Linkage disequilibrium (LD) between the markers in the QTL regions was computed using Lewontin’s normalised LD measure (D’) [15] and estimated with Haploview 4.2 [16].

To see if QTL regions overlapped with QTL regions for other traits, QTL mapping was also performed for the following traits: milk yield, fat yield, protein yield, fat content, protein content, somatic cell count, udder depth, rear udder height, fore udder attachment, locomotion, body depth, chest width, milking speed, udder support, rear teat placement, rear leg side view, stature, rump angle, rump width, front teat placement, front teat length, temperament, angularity, rear leg rear view, foot angle, direct calving ease, maternal calving ease, direct stillbirth, maternal stillbirth, interval from calving to first insemination, longevity, and clinical mastitis.

### QTL status prediction

**Figure 1:**
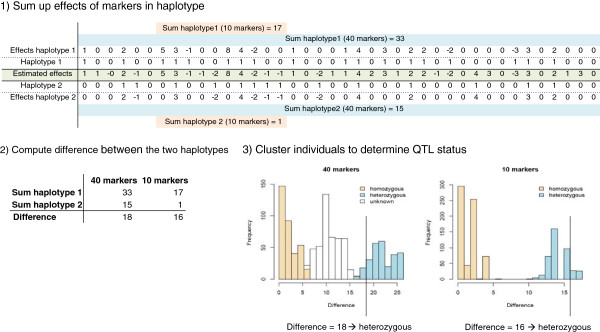
QTL status prediction.

QTL status was determined for all individuals in the QTL mapping analyses. In addition, for 33 bulls not included in the 50 K QTL mapping dataset, 50 K genotypes from Eurogenomics [17] were used to infer their QTL status, as described in [14], so that we could include them in the concordance analysis. The procedure to determine the QTL status of an individual is summarised in Figure 1. For each of the selected QTL regions, the marker effects estimated during QTL mapping were used to infer the QTL status as follows. First, genotypes were phased to define haplotypes, using DagPhase [18], while accounting for family structure. For each of the two haplotypes of an individual, a haplotype effect *H* was estimated based on a summation of estimated marker effects ${\hat{a}}_{k}$: $H=\sum k*{\hat{a}}_{k}$. This was done either for all markers in the QTL region, or for the 10, 20 or 30 adjacent markers with the highest ∑p in the region. Subsequently, the difference between the estimated effects of the two haplotypes was used to determine if an individual was homozygous or heterozygous: if both haplotypes had similar effects, the individual was homozygous, while if the difference between the two haplotypes was substantially larger than 0, the individual was heterozygous. Individuals were grouped based on the absolute value of the difference between two estimated haplotype effects using the following posterior around methods (PAM) [19], as implemented in the fpc R-package [20]:

1. *k* medoids were randomly selected from the data.

2. All non-medoids were assigned to the closest medoid. The costs of configuration when medoid and data point are switched were calculated using Euclidean distance.

3. The configuration with the lowest cost was selected.

4. Steps 2 and 3 were repeated until the medoids remained equal.

The number of clusters (*k*) was estimated based on the optimum average silhouette [21], using two, three, or four groups. The QTL status of animals in the cluster with the lowest haplotype difference was denoted homozygous, and that of animals in the cluster with the highest difference was denoted heterozygous. If more than two clusters were present, the QTL status of animals in the other clusters was denoted unknown.

### Concordance analysis

The concordance analysis compares the estimated QTL status with the genotype of polymorphisms present in the QTL region across individuals. Genotypes of 71 Holstein bulls for polymorphisms detected in the 1000 Bull Genomes project [22] were used for the concordance analysis. For each QTL, a list of polymorphisms present in the QTL region and the corresponding genotypes of the individuals were obtained. Polymorphisms included both SNPs and indels. Regardless of the interval size used for status prediction, the initially detected 40-marker QTL intervals were considered for the concordance analysis. Subsequently, the status of the polymorphisms was compared with the QTL status across individuals. Polymorphisms were only compared with the QTL status of a certain individual if the genotype quality score of the sequence in that individual was equal to 20 or higher. The probability of polymorphisms being concordant by chance was calculated following Ron et al. [3]:

$$p_{c}=\overset{1}{\underset{0}{\int }}\left(2{\left[p\left(1-p\right)\right]}^{n}{\left[1-2p\left(1-p\right)\right]}^{m}\mathrm{dp}\right),$$

where *p* is the allele frequency of the reference allele, and *n* and *m* the number of heterozygous and homozygous individuals, respectively.

A polymorphism was considered concordant with a QTL if:

1. at least 90% of the individuals were either homozygous for both the polymorphism and the QTL or heterozygous for both the polymorphism and the QTL,

2. its genotype quality score was equal to 20 or higher for at least five homozygous and five heterozygous individuals,

3. and its probability of concordance by chance (*p*_*c*_) was lower than 1 divided by the total number of polymorphisms present in the QTL region.

For the concordant polymorphisms, annotations were obtained using the “variant effect predictor” application from Ensembl [23] to generate the functional consequences of polymorphisms.

## Results

### QTL mapping

**Figure 2:**
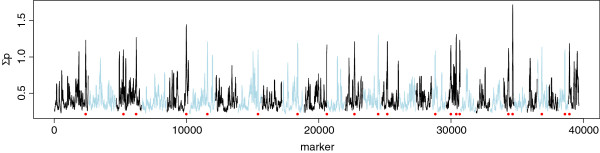
**Sum of posterior inclusion probability (∑p) across the genome.** Selected QTL are indicated with red dots.

QTL for RLSV were detected on chromosomes 1, 3, 5, 6, 8, 10, 11, 13, 14, 15, 18, 19, 23, 26, 28, and 29. Figure 2 shows the distribution of ∑p along the genome and the selected QTL regions. The 20 selected QTL regions with their location and ∑p are in Table 1. The ∑p for the QTL regions ranged from 1.08 to 1.72 when using 40-marker intervals. Reducing the size of the interval to 30, 20 or 10 markers changed the order of intervals. When intervals of 30 markers were considered, the four largest QTL remained the same but the ranking of most other QTL changed. With an interval size of 10 markers, the ranking was completely different, with the exception of QTL 3.

**Table 1:** Selected regions and their posterior inclusion probabilities, using different interval sizes

	***40 markers***				***30 markers***			***20 markers***			***10 markers***		
**QTL**	**chr**	**start**	**end**	**∑p**_**40**_	**rank**	**∑p**_**30**_	**∑p**_**30**_**/∑p**_**40**_	**rank**	**∑p**_**20**_	**∑p**_**20**_**/∑p**_**40**_	**rank**	**∑p**_**10**_	**∑p**_**10**_**/∑p**_**40**_
1	23	46.1	48.2	1.72	1	1.55	0.90	1	1.09	0.63	7	0.75	0.44
2	5	109.6	111.7	1.45	2	1.21	0.83	7	0.93	0.64	24	0.57	0.39
3	19	47.7	49.8	1.31	3	1.19	0.91	2	1.02	0.78	3	0.83	0.63
4	14	66.3	69.4	1.31	4	1.15	0.88	4	0.98	0.75	9	0.73	0.56
5	3	98.6	100.7	1.27	12	0.99	0.78	8	0.90	0.71	15	0.62	0.49
6	19	60.3	62.5	1.23	7	1.09	0.89	18	0.79	0.64	18	0.60	0.49
7	1	146.6	148.2	1.23	15	0.97	0.79	13	0.85	0.69	23	0.57	0.46
8	15	29.5	31.3	1.22	8	1.08	0.89	6	0.94	0.77	35	0.50	0.41
9	13	46.5	48.4	1.21	16	0.96	0.79	31	0.67	0.55	40	0.46	0.38
10	6	87.2	90.3	1.21	10	1.01	0.83	25	0.74	0.61	32	0.51	0.42
11	10	74.9	78.2	1.19	5	1.11	0.93	3	1.02	0.86	1	0.85	0.71
12	29	6.6	9.1	1.19	9	1.01	0.85	9	0.90	0.76	14	0.62	0.52
13	11	104.6	107.0	1.17	6	1.09	0.93	5	0.97	0.83	2	0.84	0.72
14	19	23.9	26.0	1.16	14	0.97	0.84	23	0.76	0.66	19	0.59	0.51
15	26	21.8	24.3	1.14	21	0.94	0.82	17	0.81	0.71	16	0.61	0.54
16	23	24.2	27.8	1.12	23	0.93	0.83	22	0.76	0.68	21	0.57	0.51
17	28	30.8	33.9	1.10	11	0.99	0.90	10	0.89	0.81	4	0.78	0.71
18	8	96.5	98.8	1.10	22	0.93	0.85	28	0.71	0.65	26	0.55	0.50
19	3	40.6	43.2	1.10	20	0.95	0.86	14	0.85	0.77	8	0.74	0.67
20	18	14.0	16.8	1.08	24	0.93	0.86	20	0.77	0.71	12	0.63	0.58

The 20 QTL with the largest sum of posterior inclusion probabilities (∑p) and their location in Mb, ranked based on their ∑p considering an interval size of 40 markers (~2.5 Mb), and their rank and ∑p if smaller intervals of 30 (~1.9 Mb), 20 (~1.3 Mb) or 10 (~0.6 Mb) are used.

### Status prediction

There was a large variation in the distribution of the estimated haplotype differences. When the complete 40-marker interval used for QTL mapping was taken into account for QTL status prediction, there was no visible separation between homozygous and heterozygous individuals and thus, it was not possible to predict QTL status accurately for most QTL and individuals. With an interval size of 40 markers, individuals were successfully separated in two distinct groups for only three of the 20 QTL, QTL 11, 15, and 19. For three other QTL, QTL 3, 13, and 20, individuals were grouped in more than two groups, thus putting a group with unknown status between the homozygous and heterozygous individuals. Reducing the interval size improved the status derivation: with 10-marker intervals, a separation between homozygous and heterozygous individuals could be observed for most QTL. For half of the QTL, i.e. QTL 4, 6, 9, 11, 12, 14, 15, 18, 19 and 20, two clearly separated clusters were obtained, while for QTL 1, 3, 7, 13 and 17, individuals were clustered in more than two groups. However, for QTL 2, 5, 8, 10 and 16, distinguishing between homozygous and heterozygous individuals remained difficult. Therefore, these QTL were not used for subsequent concordance analysis. For the QTL with inferred status, the numbers of individuals that were predicted to be homozygous, heterozygous and unknown for the QTL are in Table 2.

**Table 2:** QTL status prediction counts

**QTL**	**Homozygous**	**Heterozygous**	**Unknown**	**n**_**poly**_	**p**_**c**_
1	35	6	30	20 486	4.28 × 10^-24^
3	29	15	27	17 365	4.70 × 10^-23^
4	42	29	0	21 333	4.28 × 10^-35^
6	37	34	0	23 499	1.95 × 10^-33^
7	24	29	18	13 858	7.86 × 10^-24^
9	41	30	0	12 124	9.06 × 10^-35^
11	35	36	0	29 105	9.66 × 10^-33^
12	41	30	0	27 411	9.06 × 10^-35^
13	26	20	25	28 541	1.25 × 10^-22^
14	34	37	0	22 154	2.20 × 10^-32^
15	37	34	0	17 454	1.95 × 10^-33^
17	17	20	34	25 321	6.30 × 10^-17^
18	42	29	0	15 746	4.28 × 10^-35^
19	36	35	0	27 747	4.32 × 10^-33^
20	46	25	0	22 771	2.22 × 10^-36^

For the QTL for which QTL statuses could be inferred, the number of homozygous, heterozygous and unknown individuals, the number of polymorphisms in the QTL region (n_poly_) and the probability of concordance by chance (p_c_).

**Figure 3:**
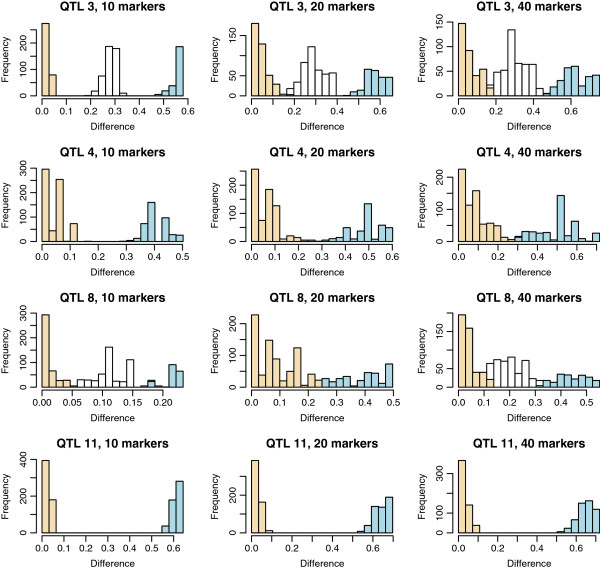
**Distribution of the absolute difference between haplotype effects, depending on interval size.** Haplotype effects were approximated by the sum of estimated marker effects for all markers in a haplotype.

**Figure 4:**
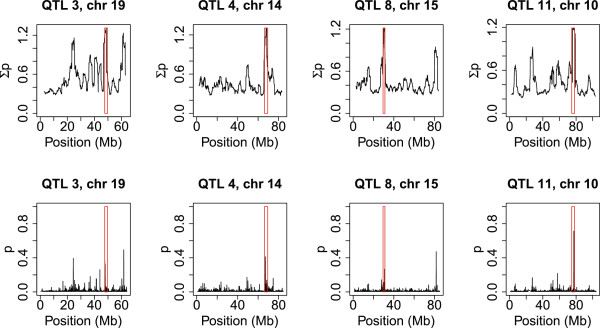
**Sum of posterior inclusion probability (∑p) and posterior inclusion probability per marker (p) for QTL 3, 4, 8, and 11.** QTL regions are indicated with red rectangles.

Figure 3 shows the status prediction with interval sizes of 10, 20 or 40 adjacent markers for QTL 3, 4, 8 and 11. For QTL 11, a separation between homozygous and heterozygous individuals was observed with a 40-marker interval. Decreasing the interval size to 20 markers improved the distribution for QTL 3 and 4, and a further decrease to 10 markers resulted in clear separation between homozygous and heterozygous individuals for QTL 4, while for QTL 3, individuals were divided in three groups, homozygous, heterozygous and a middle group with an undetermined status. For QTL 8, no separation was observed, regardless of the interval size. For QTL 3, 4, 8 and 11, Figure 4 shows both the ∑p and the posterior inclusion probability for each SNP. For QTL 11, there was one major peak in the interval, while several peaks were observed for QTL 3, 4 and 8.

### Concordance analysis

The results of the concordance analysis for the 15 QTL for which status could be inferred are in Table 3. The number of concordant polymorphisms was on average equal to 70 and was generally lower for QTL for which the individuals were clustered in two groups than for QTL with more than two clusters, for which, on average, 202 concordant polymorphisms were found.

**Table 3:** Concordant polymorphisms for QTL that were clustered in two or more than two groups

**QTL**	**indiv**	**conc**	**intg**	**intr**	**down**	**up**	**other**	**full**	**genes**
*2 groups*									
4	58	42	4	37	0	1	0	0	*VPS13B*, *OSR2*
6	64	15	0	12	3	0	0	0	*MAP2K6*
9	59	8	0	7	1	0	0	1	*ADARB2*, miRNA
11	65	27	24	0	3	0	0	0	*5S rRNA*
12	43	3	3	0	0	0	0	0	-
14	58	102	100	0	0	2	0	22	*RAP1GAP2*
15	59	340	115	197	0	28	0	6	*BTRC*, *LBX1*
18	59	63	63	0	0	0	0	1	-
19	59	35	0	35	0	0	0	0	*COL11A1*
20	59	65	65	0	0	0	0	0	-
*>2 groups*									
1	40	327	265	42	4	12	4	0	*SLC35B3*, *EEF1E1*, *BLOC1S5*, *TXNDC5*, *5S rRNA*, *BMP6*, *U6*, *DSP*, *RIOK1*, *CAGE1*, *SSR1*, *RREB1*
3	37	52	40	8	2	1	1	0	*TANC2*, *ACE3*, *SCN4A*, *ICAM2*, *ERN1*, miRNA
7	46	411	197	126	39	47	2	0	*PCBP3*, *5S rRNA*
13	34	31	7	21	3	0	0	3	*BRD3*, *EHMT1*, *ARRDC1*, *MRPL41*, *WDR85*, *PNPLA7*, *NELF*, *EXD3*
17	29	187	113	71	3	0	0	97	*KAT6B*, *KCNMA1*, pseudogene

indiv = number of concordant polymorphisms for the QTL used in the concordance analysis, the average number of individuals with known QTL status and sequence quality score ≥ 20; conc = number of concordant polymorphisms; intg = number of intergenic concordant polymorphisms; intr = number of intronic concordant polymorphisms; down = number of downstream concordant polymorphisms; up = number of upstream concordant polymorphisms; other = number of other concordant polymorphisms including 3′ prime variants, synonymous variant, splice variants and non-coding exon variants; full = polymorphisms in complete concordance; and names of the genes containing concordant polymorphisms.

**Figure 5:**
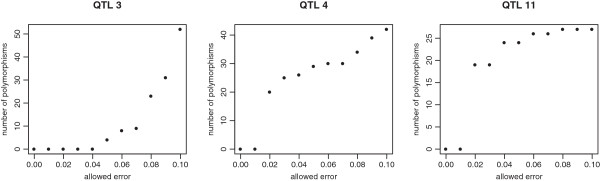
Number of concordant polymorphisms, depending on the accepted proportion of non-concordant individuals.

Because sequence errors are likely to occur, polymorphisms were considered concordant if they were concordant for at least 90% of the individuals, rather than setting a 100% concordance. If a 100% concordance had been set, the number of concordant polymorphisms would have been substantially reduced. Most QTL had no polymorphisms in complete concordance. Complete concordant polymorphisms were found only for QTL 9, 13, 14, 15 and 18. Figure 5 shows the reduction in the number of concordance polymorphisms when the threshold of allowed errors was reduced from 10% to 0% for QTL 3, 4 and 11. For QTL 3, for which the status of some of the animals was set to unknown, the number of concordant polymorphisms was reduced much more than for QTL 4 and 11 for which complete concordance was required. For QTL for which individuals were clustered in two groups, a large proportion of the concordant polymorphisms was still concordant when the error threshold was reduced to 5%, while for QTL for which individuals were clustered in more than two groups, a much lower proportion of polymorphisms remained concordant.

The number of concordant polymorphisms for the QTL for which individuals were clustered in two groups ranged from 3 for QTL 12 to 340 for QTL 15.

**Figure 6:**
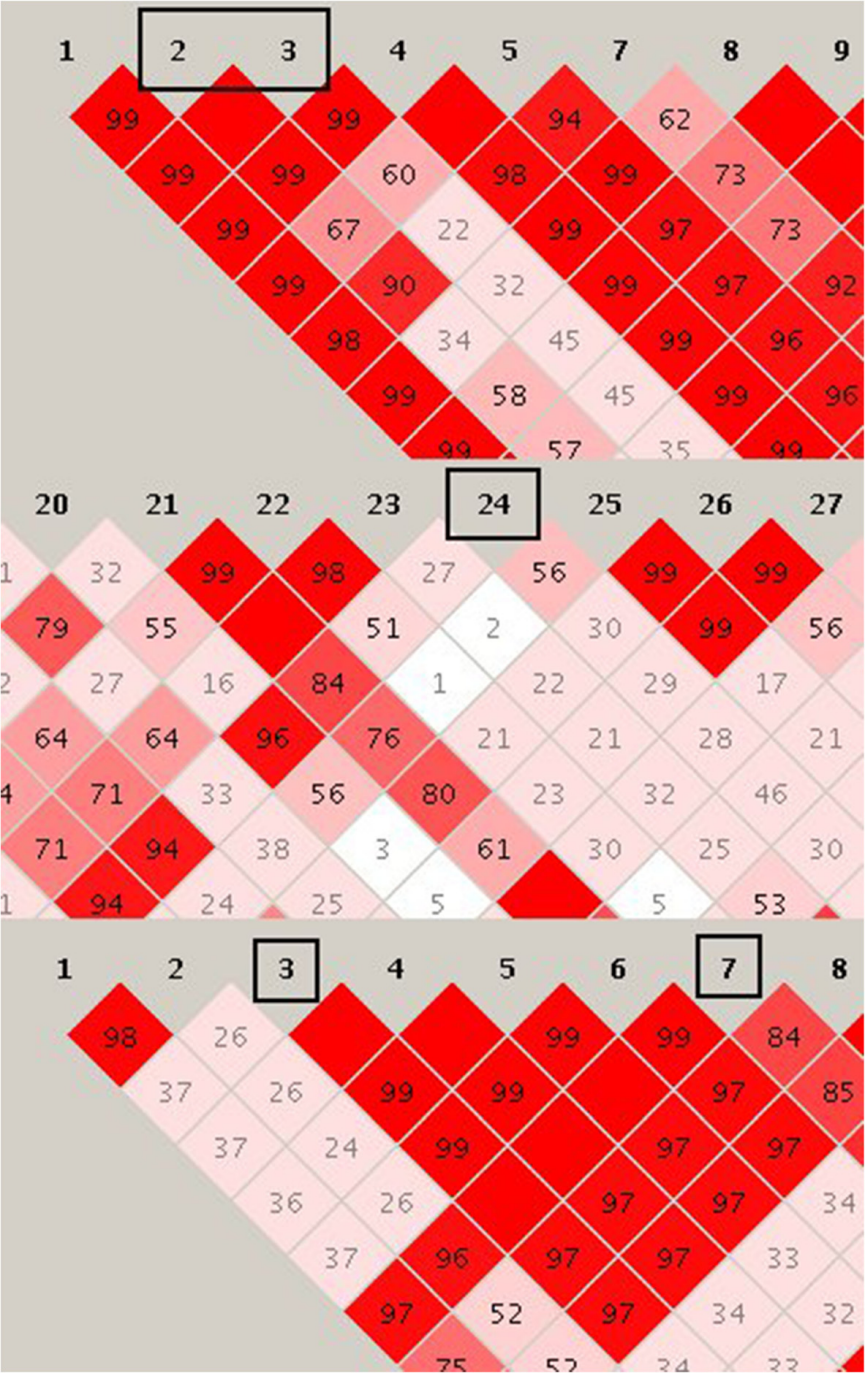
**Linkage disequilibrium (LD) plots of QTL 9, 11 and 15.** LD between markers in the QTL interval was estimated by D’; blocks containing concordant polymorphisms are indicated with black rectangles.

Figure 6 shows LD plots for QTL 9, 11 and 15. The two regions that contained concordant polymorphisms for QTL 9 were in high LD with other regions, but only in complete LD with each other. Concordant polymorphisms for QTL 11 were all located in the same region, which was in low LD with other segments of the QTL region. The two blocks that contained concordant polymorphisms for QTL 15 were in complete LD with each other.

The concordant polymorphisms for QTL for which haplotype effects clustered in two groups, were located in at most two genes, while concordant polymorphisms for QTL for which effects clustered in more than two groups, were generally spread over a larger number of genes.

For QTL 4, 42 polymorphisms were in concordance, of which four were intergenic, 26 were in introns of the *VPS13B* gene, one was in an intron of the *OSR2* gene, and one was upstream of this gene. Twelve of the 15 concordant polymorphisms for QTL 6 were intronic variants of the *MAP2K6* gene, while the remaining three polymorphisms were located in the downstream region of the same gene. Of the eight concordant polymorphisms found for QTL 9, seven were intronic variants of the *ADARB2* gene and one polymorphism was located downstream of a microRNA gene. For QTL 12, only three intergenic polymorphisms were in concordance with the QTL. The number of comparisons that could be made for two of these variants was limited due to the low quality of the sequence at these positions for most individuals. Almost all of the 102 concordant polymorphisms for QTL 14 were intergenic, except for two polymorphisms located upstream of the *RAP1GAP2* gene. For QTL 15, 340 polymorphisms were concordant, of which 115 were intergenic, one was upstream of the *LBX1* gene, 197 were in introns of the *BTRC* gene, and 27 were upstream of this gene. All 63 and 65 concordant polymorphisms for QTL 18 and 20, respectively, were intergenic. The 35 concordant polymorphisms for QTL 19 were all intronic variants of the *COL11A1* gene.

The concordant polymorphisms for QTL 1, 3 and 13 were scattered over a large number of genes. QTL 7 had the largest number of concordant polymorphisms, i.e. 441, of which 197 were intergenic, two were in non-coding exons of a 5S rRNA, 39 and 13 were respectively downstream and upstream variants of the same 5S rRNA, 196 were in introns of the *PCB3* gene, and 34 were upstream variants of this gene. In total, 187 polymorphisms were in concordance with QTL 17. Of these polymorphisms, 113 were intergenic, three were downstream variants of a pseudogene, 65 were intronic variants of the *KAT6B* gene and six were intronic variants of the *KCNMA1* gene.

### Associations with other traits

**Figure 7:**
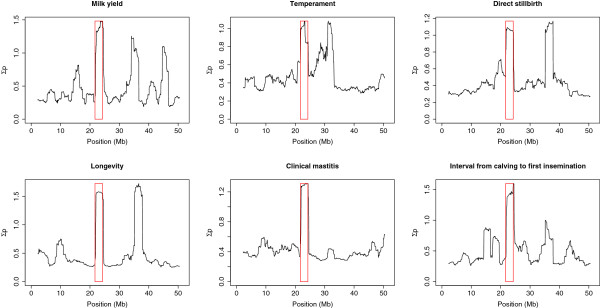
**Association of QTL 15 with other traits.** Sum of posterior inclusion probabilities (∑p) on chromosome 26 for milk yield, temperament, vitality at birth, longevity, clinical mastitis and interval from calving to first insemination with the QTL detected for rear leg side view indicated with a red rectangle.

Most of the QTL detected for RLSV also showed peaks in ∑p for several other traits. Table 4 shows, for each QTL region, the traits that had a ∑p of at least 0.8. In particular, in the intervals that contained QTL 10 and 15, peaks in ∑p were observed for a large variety of traits. QTL 15 was, for example, also associated with milk yield, protein yield, fat content, protein content, somatic cell count, udder depth, udder support, angularity, maternal calving ease, longevity, clinical mastitis, and interval from calving to first insemination. Figure 7 shows the association between QTL 15 and several traits.

**Table 4:** Association of QTL regions for rear leg side view with other traits

**QTL**	**Traits with ∑p ≥ 0.8**
1	Locomotion, milking speed, rump angle
2	Locomotion, stature, angularity, foot angle
3	Fat content, rump angle, foot angle
4	Milk yield, fat content, protein content, somatic cell count, rear udder height, udder support, rear teat placement, rump angle
5	Fat content
6	Stature, rump angle, rump width
7	Somatic cell count, rear teat placement
8	Front teat length
9	Protein content, rump width
10	Milk yield, protein yield, fat content, protein content, somatic cell count, udder depth, udder support, angularity, maternal calving ease, longevity, clinical mastitis, interval from calving to first insemination
11	Locomotion, rear leg rear view
12	Protein content, rump width, front teat length
13	Chest width, rump angle
14	Locomotion, foot angle
15	Milk yield, fat yield, protein yield, fat content, protein content, somatic cell count, rear udder height, rump width, temperament, direct stillbirth, longevity, clinical mastitis, interval from calving to first insemination
16	Body depth, front teat length, maternal calving ease
17	Locomotion, chest width, stature, direct stillbirth
18	-
19	Locomotion, rear leg rear view
20	Fat yield, maternal calving ease

Traits for which the sum of posterior inclusion probabilities (∑p) in a QTL region equalled at least 0.8.

## Discussion

### Concordance analysis

For 15 of the 20 QTL regions analysed, we were able to strongly reduce the number of candidate mutations by applying concordance analysis. For eight of these QTL, the regions were narrowed down to polymorphisms located in one or two genes.

For most of the detected QTL, the distribution of the haplotype differences did not show a clear grouping when all markers in the QTL interval were used to compute the haplotype effects. This was especially the case for the QTL with a larger effect. All 20 QTL had a ∑p larger than 1. ∑p can be larger than 1 because several markers can together explain a QTL, and are thus simultaneously included in the model, or because more than one causative mutation may be present. It is likely that the largest QTL are affected by multiple mutations in the same region rather than by a single mutation. If these mutations have approximately the same effect, the distributions of estimated marker effects will overlap and it is not possible to distinguish between heterozygous individuals with different mutations, which can explain the difficulty in status prediction. When a smaller interval is used to infer the QTL status, fewer mutations will be located in the interval. As a consequence, QTL status could be predicted for a much larger number of QTL when a smaller interval of 10 markers was used. The ∑p of these intervals was much lower than the ∑p for the complete interval, especially for the QTL for which there were difficulties with status prediction using the complete interval. For example, the highest ∑p was equal to 1.72 when the 40-marker interval (QTL 1) was used, but dropped to 0.75 when only 10 markers were used. Although using the smaller interval size made it possible to infer the QTL status for a larger proportion of the QTL, this approach may ignore a major part of the QTL by focussing on a single mutation. A more detailed analysis is required to determine whether there are indeed multiple mutations present in these regions and to disentangle their effects. For example, by imputing SNPs to the sequence level for the complete QTL detection design, followed by an association study using the imputed sequences. Specifically, multiple causal variants in a QTL region can be tested using a multiple SNP association model in this region.

Alternatively, it is possible to predict the QTL status of sires using progeny data [6] but this requires data of a sufficiently large number of progeny. For most sires in our dataset, the amount of available data for progeny was not sufficient to accurately derive the QTL status. Thus, it would only be possible to predict the QTL status for a limited number of individuals, which would be too low for a large-scale concordance analysis. Furthermore, if the difficulties in status prediction are indeed due to the presence of multiple QTL in the same interval, then this will cause the same problems in status prediction using the granddaughter design.

Concordance analysis could only be applied for the 15 QTL for which QTL status could be inferred. The number of concordant polymorphisms and the number of genes in which these polymorphisms were located varied widely. For the QTL for which the status could only be accurately inferred for part of the sequenced individuals, the concordant polymorphisms were spread over more genes than for the QTL for which the status could be inferred for all individuals. This shows that a large number of records is necessary to narrow a region down to one or two genes using concordance analysis. Apart from this, the success of concordance analysis also depends on the LD between polymorphisms. Nearby polymorphisms can be in complete LD and, as a consequence, several polymorphisms other than the causative mutation may be concordant with the QTL. The concordance analysis seemed to be able to distinguish between parts of the genome with high levels of LD. For example, the blocks that contained concordant polymorphisms for QTL 15 were in complete LD with each other. Although they were almost in complete LD (99%) with the blocks in between, concordant polymorphisms were only found in the blocks that were in complete LD with each other. This suggests that with a sufficient number of sequences, concordance analysis can distinguish between polymorphisms that are in high but incomplete LD.

Since both status prediction and sequencing data can contain errors, we allowed for some non-concordant animals. The threshold of allowed non-concordant individuals was set arbitrarily to 10%. When this threshold was reduced, the number of concordant polymorphisms decreased. This decrease was much greater for QTL with more than two clusters than for QTL with two clusters. For the latter QTL, a lower number of comparisons could be made because the QTL status of the middle group was unknown.

### Annotations

Concordant polymorphisms for QTL 4 were intergenic or located in the genes *VPS13B* and *OSR2*. In humans, mutations in *VPS13B* cause the Cohen syndrome, for which symptoms include mental retardation, facial dysmorphism, microcephaly, retinal dystrophy, truncal obesity, joint laxity and intermittent neutropenia [24]. In mice, *ORS2* is involved in craniofacial, limb and kidney development [25], palatal growth and patterning [26], and synovial joint formation [27]. Its role in limb development makes it a good candidate gene for RLSV.

All concordant polymorphisms for QTL 6 were located in the *MAP2K6* gene, which is expressed in the skeletal muscle, heart, liver and pancreas in mice [28]. In mice, effects attributed to a mutation in this gene include a dwarf phenotype, caused by reduced chondrocyte proliferation, inhibition of hypertrophic chondrocyte differentiation and a delay in the formation of primary and secondary ossification centres [29].

Only eight polymorphisms were concordant with QTL 9, of which one was located downstream of a microRNA and seven were in introns of the *ADARB2* gene, an RNA editing gene associated with longevity in both humans and *C. elegans*[30]. Although RLSV is correlated with longevity in cattle [7] and several of the QTL regions did show peaks in ∑p for longevity, this is not the case for QTL 9.

Concordant polymorphisms for QTL 11 were intergenic, except for three polymorphisms that were located in the downstream region of the 5S rRNA, a part of the ribosome that is required for normal translation in most ribosomes but with no known precise function [31].

For the QTL with two clusters, the largest number of concordant polymorphisms was found for QTL 15, i.e. 340, of which 115 were intergenic variants, 197 were in introns of the *BTRC* gene, 27 were upstream variants of this gene and one was an upstream variant of the *LBX1* gene. In mice, mutations in the *BTRC* gene are reported to affect spermatogenesis [32], mammary gland development [33], tumorigenesis [33] and retinal development [34]. Both *BTRC*[35,36] and *LBX1*[36] have been associated with split-hand/split-foot malformations in humans. Furthermore, *LBX1* is involved in limb development in mice [37,38], thus it is a good candidate gene for a QTL involved in bovine leg conformation. In addition, in mice the gene *LBX1* is reported to play a role in neural tube development [39], heart development [40], and central respiratory rhythmogenesis [41]. Thus, a wide range of effects have been identified for mutations in these genes in humans and mice. Interestingly, the QTL region detected for RLSV also affected a large number of other traits in dairy cattle, including longevity, confirmation, milk production, clinical mastitis and temperament.

All concordant polymorphisms of QTL 19 were located in introns of the *COL11A1* gene. In mice, mutations in *COL11A1* result in chondrodysplasia, which is characterized by various skeletal defects [42-44], including a rotated distal portion of the hind limbs [42]. Other reported effects in mice relate to tendon development [45], myocardial morphogenesis, and heart valve development [46]. Furthermore, mutations in the gene *COL11A1* have been associated with Marshall [47] and Stickler [48] syndromes in humans, which include skeletal abnormalities. Thus, with skeletal effects in both humans and mice, *COL11A1* is a good candidate gene for a QTL involved in RSLV.

For most of the QTL for which the status prediction resulted in more than two clusters, the concordance analysis resulted in concordant polymorphisms in a large number of genes. Only for QTL 7 and 17, did the concordance analysis narrow the regions down to specific genes. Concordant polymorphisms for QTL 7 were either intergenic, or located in a 5S rRNA gene or in the *PCBP3* gene. Molecular functions attributed to *PCBP3* include DNA binding and RNA binding [49]. For QTL 17, concordant polymorphisms were intergenic, located in the downstream region of a pseudogene, or intronic variants of the *KAT6B* and *KCNMA1* genes. In mice, reduced expression of *KAT6B* results in developmental anomalies of the skeleton and brain [50]. In humans, *KAT6B* has been associated with Ohdo syndrome for which symptoms include skeletal, facial, cardiac and dental abnormalities [51] and with genitopatellar syndrome [52], a skeletal dysplasia. In mice, mutations in the *KCNMA1* gene cause cerebellar dysfunction, abnormal locomotion, and deficient motor coordination [53]. QTL 17 is also associated with locomotion.

Concordant polymorphisms for QTL 1 were present in 12 genes, including 15 intronic variants of the *BMP6* gene, which is involved in cartilage and bone formation [54]. Six genes with polymorphisms concordant with QTL 3 were identified. Of these six genes, *SCN4A* is known to cause muscle weakness in mice [55] and humans [56]. The known functions of the eight genes that contained concordant polymorphisms for QTL 13 are not clearly related to RLSV, except for *EHMT1*, which is associated with Kleefstra syndrome in humans [57]. Although limb abnormalities are not part of the main characteristics of this syndrome, they are present in some patients [57].

Concordant polymorphisms were mainly located in the non-coding regions of the genome. This is also the case for the majority of disease- and trait-associated variants identified in human GWAS and it has been suggested that such non-coding variants are involved in transcriptional regulatory mechanisms [58].

## Conclusions

We were able to perform concordance analysis for 15 of the 20 regions that were most likely to contain QTL for RLSV. For those regions, we could reduce the number of candidate mutations. For some QTL, the concordant analyses narrowed the identified region down to a limited number of genes. Some of these genes are known for their role in limb development, skeletal development in humans and mice, or other effects related to RLSV. Thus, mutations in these genes are good candidates for QTN that affect RLSV.

## Competing interests

The authors declare that they have no competing interests.

## Authors’ contributions

IB, DB, MSL and DR designed the study. IB, DB and MSL carried out the study and drafted the manuscript. SF generated and provided phased data. MB and SR generated and provided annotations. All authors read and approved the final manuscript.
